# Short‐Chain Fatty Acids as Potential Mediators of NSAIDs’ Effects on Arthritis Pain Relief

**DOI:** 10.1155/prm/9190926

**Published:** 2026-06-26

**Authors:** Xuesong Wang, Hao Meng, Baoshan Yuan, Dongsong Liu, Yongning Dong, Feng Zhang, Hong Cao, Dan Li, Ju Yang, Jing Sun, Yingyu Wang, Bingqian Chen, Jiai Yan

**Affiliations:** ^1^ Department of Clinical Nutrition, Affiliated Hospital of Jiangnan University, Wuxi, Jiangsu, 214122, China, jiangnan.edu.cn; ^2^ Department of Orthopaedics, Jianhu County People’s Hospital, Yancheng, 224700, China; ^3^ School of Medicine, Nantong University, Nantong, Jiangsu, 226019, China, ntu.edu.cn; ^4^ Nutrition and Intestinal Microecology Laboratory, Institute of Future Food Technology, Yixing, Jiangsu, 214200, China; ^5^ Department of Orthopaedics, Changshu Hospital Affiliated to Soochow University, First Peoples’ Hospital of Changshu City, Changshu, Jiangsu, 215500, China

**Keywords:** gut microbiome, nonsteroidal anti-inflammatory drugs, osteoarthritis, pain, short-chain fatty acids

## Abstract

**Background:**

Nonsteroidal anti‐inflammatory drugs (NSAIDs) are the preeminent choice for treating osteoarthritis (OA) and have demonstrated the ability to regulate the gut microbiome. This study aims to explore the possibility that pharmacologically modified microbiome plays a role in improvement of arthritis.

**Method:**

Rats with the right knee joint subjected to anterior cruciate ligament transection (ACLT) surgery were randomly allocated to eight groups. Four weeks after the procedure, the rats were given an intervention with saline (NS), celecoxib (CE), diclofenac (DF), and tramadol (TM). The rats were executed after 6 weeks of intervention, serum was used to evaluate the levels of inflammation and pain‐related factors (IL‐1β, IL‐6, TNF‐α, LPS, CGRP, and NGF), rat joints were used to assess local inflammation in the joints (MyD88) and the effect of the drug on the cartilage (Micro CT, Safranin O–Fast Green stain), Von Frey fibrous filaments were used to assess the sensation of pain in rats, and the differences in feces for the gut microbiome and short‐chain fatty acids (SCFAs) were compared by 16S rRNA sequencing and gas chromatographic analyses.

**Result:**

Rats treated with CE, DF, and TM experienced significant relief from joint pain, but the analgesic effect of CE and DF was significantly weakened after the combined application of antibiotics, while the effect of TM on pain remained after the application of antibiotics. The expression of MyD88 in the articular cartilage was somewhat reduced by CE and DF. Additionally, the gas analysis showed a significant increase in the SCFA content in the feces of the drug‐intervention group.

**Conclusion:**

NSAID‐induced changes in gut microbiota structure and the subsequent increase in SCFA production may contribute to their pain‐alleviating effects in rats. Although to a lesser extent, NSAIDs can also slightly reduce localized inflammation in the joints.

## 1. Introduction

Osteoarthritis (OA) is the leading cause of disability in adults aged 60 years and over, with prevalence increasing dramatically with age [1]. According to recent studies, 14.8% of the people worldwide suffers from OA, and as the population ages, this percentage is predicted to become much higher, placing a severe strain on both global health and the economy [2]. There are currently no effective therapies for OA, which is a degenerative condition marked by the loss of cartilage in the joints. Patients with OA frequently have chronic pain and joint dysfunction, and many of them rely on long‐term oral painkillers, such as nonsteroidal anti‐inflammatory drugs (NSAIDs), to relieve pain, which significantly lowers their quality of life.

Inflammation is now considered to have a more significant impact in the development of this condition, which was previously thought to be more of a wear‐and‐tear disease [3]. Low‐grade inflammation mediated by innate intrinsic immunity has been proved to play a key role in the progression of OA [4]. The improvement of OA had been conclusively demonstrated to be associated with suppression of inflammation [5], which had led to an increased focus on the investigation of inflammation mitigation, with the hope of regulating inflammation in order to achieve relief from OA. Due to the contact and crosstalk between the gut microbiome and the human immune system, the function of the gut microbiome in OA had gained interest [6]. Gut microbiome, defined as the collection of microorganisms localized in the intestines, is an essential component of the human immune system that aids in the development and maturity of the immune system as well as the body’s resistance to infections [7]. With the deepening of research, there was mounting proof that the chemicals produced by the gut microbiome play an important role in the development of OA [8–10]. For OA, the gut microbiome became a potential therapeutic target.

OA was commonly treated with NSAIDs. They had analgesic and anti‐inflammatory properties because they inhibited the cyclooxygenase from producing prostaglandins [11]. Although the cartilage‐protective effects of this class of drugs were controversial [12–14], their specific pain‐relieving effects had led to their widespread usage in the treatment of OA. However, as the critical role of inflammation in the progression of OA has been increasingly recognized, the inhibitory effect on inflammation in improving the process of OA deserves to be reexamined. Notably, research has shown that NSAIDs not only inhibit the activity of cyclooxygenase but also, and to varying degrees, alter the composition of the gut microbiome [15]. In recent years, the impact of NSAIDs on gut microbes has been more specifically characterized with the development of sequencing technologies, but the contribution of drug‐induced disruption of the gut microbiome to disease progression and the efficacy of drug treatments has rarely been reported.

Therefore, this study investigated the effects of different types of NSAIDs on gut microbiome structure and OA. Considering the differential effects of different types of drugs on gut microbiota, we chose two different NSAIDs as representatives, hoping to identify common effects on gut microbiota and to explore the effects of gut microbiota on OA from the differences. Our experimental results may provide a deeper insight into the role of the gut microbiome in OA and provide a new theoretical basis and practical guidance for the clinical treatment of OA.

## 2. Materials and Methods

### 2.1. Animals

Five‐week‐old male SD rats weighing 176–200 g were purchased from Vital River (Zhejiang, China), and 3 rats were housed in a cage in a standard laboratory environment, where the rats had free access to standard rat food and water. Each SD rat was given 7 days to adapt to the laboratory environment before experiments were performed. The study was approved by the Ethics Committee of Wuxi Drug Safety Inspection and Testing Center (2022WXYJ10‐01).

### 2.2. Arthritis Model

The anterior cruciate ligament (ACL) was surgically damaged in SD rats to establish the ACL transection (ACLT) [16]. Briefly, the joint capsule near the medial aspect of the patellar tendon was incised, and then the knee was flexed, and the ACL was exposed and dissociated, making the joint unstable.

### 2.3. Pseudo‐Aseptic Treatment

According to Cho [17], the gut microbiota in rats was depleted by drinking combined antibiotic water containing metronidazole (1 g/L), ampicillin (1 g/L), neomycin sulfate (1 g/L), and vancomycin (0.5 g/L).

### 2.4. Study Design

**FIGURE 1:**
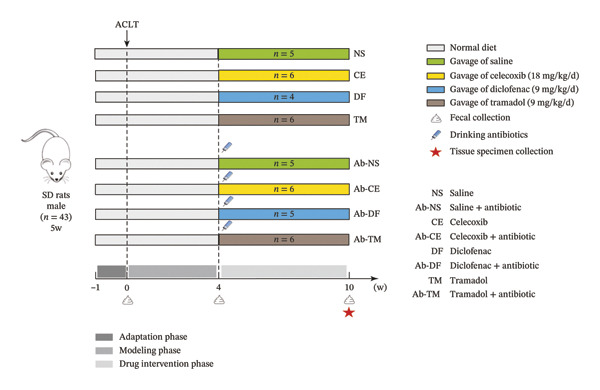
Flow diagram of the experiment.

All rats underwent surgical disconnection of the ACL after a week of adaptation period to induce OA, and drug intervention was started after 4 weeks of surgery. Rats requiring pseudo sterile modeling were given antibiotic water at the same time of drug intervention, and all rats were sacrificed after 6 weeks of drug intervention (Figure 1). The groups were as follows: the saline (NS) group: received daily NS gavage, the NS + antibiotic group (Ab‐NS): received daily NS gavage with antibiotic administration in water. The celecoxib (CE) group: received daily CE gavage, the CE + antibiotic group (Ab‐CE): received daily CE gavage with antibiotic administration in water. The diclofenac (DF) group: received daily DF gavage, the DF + antibiotics group (Ab‐DF): received daily DF gavage along with antibiotic administration in drinking water. The tramadol (TM) group: received daily TM gavage, the TM + antibiotics group (Ab‐TM): received daily TM gavage along with antibiotic administration in drinking water. Based on effective and safe doses reported in rodent studies of arthritis and pain, together with their clinically relevant equivalent doses [18–20], the concentrations used were as follows: CE at 18 mg/kg/day, DF at 9 mg/kg/day, and TM at 9 mg/kg/day.

### 2.5. Behavioral Testing for Pain

Referring to a modification of Barbosa’s [16] method, the paw withdrawal threshold (PWT) of rats was tested to assess pain sensation in rats. Rats were placed in cages with a metal mesh on the bottom for 20 min to adapt to the test environment (stop exploratory behavior and remain stationary). Von Frey fiber wires (North Coast Medical, Inc., Gilroy, CA, USA) were applied to the plantar area until the fiber wire was bent and held for 3 s. If there was a positive response (i.e., withdrawal of the hind paw, rocking, or licking), the next lower intensity fiber filament was used; if there were no responses, the next higher intensity filament was used until a positive response occurred. The stimulus intensity at which the paw was retracted was recognized as the mechanical threshold. Measurements were repeated 3 times for each rat, with an interruption of at least 20 min between each measurement. The average of the 3 measurements was recorded, and the data were expressed in grams. The testing was conducted in strict accordance with a single‐blind design, wherein the testers remained unaware of the animal grouping allocations.

### 2.6. Histopathological Parameters

Joint tissues of the knee were fixed in 4% paraformaldehyde and then decalcified in 10% EDTA for 4 weeks. The samples were then embedded in paraffin and serially sectioned (4‐μm thickness) in sagittal position along the medial compartment of the knee joint. Safranin O–Fast Green staining was used to visualize proteoglycans in the articular cartilage to assess and quantify the damage to the rat knee joint based on the Mankin score.

### 2.7. Immunohistochemistry

The 4‐μm paraffin sections were baked at 60°C for 60 min, deparaffinized in xylene and a descending gradient ethanol solution, and then washed with deionized water. Sections were placed in sodium citrate antigen repair solution for antigen repair. Endogenous peroxidase activity was blocked by 3% H_2_O_2_, and antigenic closure was performed for 30 min using blocking solution. The sections were incubated with primary antibodies against MyD88 (1:200, Proteintech, Wuhan, Hubei, China) and MMP13 (1:300, Proteintech, Wuhan, Hubei, China), respectively, at 4°C overnight. After washed with Tris‐buffered salt solution (TBS), the sections were incubated with polymer secondary antibodies containing peroxidase‐labeled polymers for 30 min at room temperature. Finally, DAB was used as the chromogenic substrate, and these sections were counterstained with hematoxylin. The average optical density, which represents the relative expression level of the target protein in each group, was calculated with Image‐J software. A blinded protocol was employed for the histological assessments. Specifically, the joint scoring and immunohistochemical analyses were performed by different investigators who were unaware of the group allocation.

### 2.8. Micro CT

The right knee joint of rats was fixed in 4% paraformaldehyde, and the entire knee joint was scanned and reconstructed in three‐dimensional images using Micro CT (PerkinElmer, USA).

### 2.9. Serum Cytokine

All blood samples were collected through the retro‐orbital plexus vein and placed in sterile EP tubes in a 4°C refrigerator overnight. Serum was obtained by centrifugation of blood at 4°C, 3500 rpm for 10 min and stored in units of 200 μL in a −80°C refrigerator. Serum levels of lipopolysaccharide (LPS), tumor necrosis factor (TNF‐α), interleukin (IL‐1β and IL‐6), calcitonin gene–related peptide (CGRP), and nerve growth factor (NGF) were assessed using specific rat enzyme‐linked immunosorbent assay (ELISA) kits (Wuhan Yunclonal Biotechnology Co., Ltd, WuHan, China) according to the manufacturer’s instructions.

### 2.10. Detection of Short‐Chain Fatty Acids (SCFAs)

The lyophilized fecal samples were mixed with 500 uL of saturated sodium chloride, shaken until homogenized, and then acidified with 10% sulfuric acid. After that, 1000 uL of ether was added to extract the SCFAs from the sample, and then mixed well and centrifuged at 12,000 rpm at 4°C for 15 min. The supernatant was collected and added to an EP tube containing anhydrous sodium sulfate, set aside for 15 min, and then centrifuged again for 15 min under the same conditions. The supernatant after the second centrifugation was taken and analyzed using GC–MS (Thermo, USA). Standard curves were used to quantify SCFAs (acetic, propionic, butyric, isobutyric, and isovaleric acids).

### 2.11. 16S rRNA Sequencing

Microbial community genomes were extracted from all fecal samples using the E.Z.N.A. ® Soil DNA Kit (Omega Bio‐tek, Norcross, GA, US) according to the manufacturer’s instructions. The concentration and purity of DNA were checked by NanoDrop2000, and the quality of DNA extraction was checked by 1% agarose gel electrophoresis. The primers for the 16S rRNA gene were amplified using the ABI GeneAmp® 9700 PCR Thermal Cycler. Products of PCR were recovered on a 2% agarose gel, purified using the AxyPrep DNA Gel Extraction Kit (Axygen Biosciences, Union City, CA, USA), eluted with Tris‐HCl, and detected by 2% agarose electrophoresis. Quantification was performed using Qubit 4.0 (Thermo Fisher, USA).

The purified amplified fragments were used to construct illumina libraries according to the standard PCR library construction procedure. PE300 sequencing was performed using Illumina’s MiSeq platform. The original fastq data were quality controlled using fastp software, and low‐quality bases and junctions were trimmed. OTU classification was performed using Vsearch software (Version 2.22.1) based on 97% similarity, and the OTU sequences were subsequently annotated for species taxonomy using RDP classifier (Version 2.13) with the confidence threshold of 0.7. The database referenced for microbiome taxonomy annotation is Silva 138.1. The overall structure of the microbial community was analyzed using unweighted UniFrac principal coordinate analysis (PCoA). Unweighted UniFrac PCoA was used to analyze the composition of the microbial community. A linear discriminant analysis of effect sizes (LEfSe, Galaxy Version 1.0) was used to identify differences between groups.

### 2.12. Statistical Analysis

All experimental data were expressed using mean ± standard deviation (mean ± SD). Data were processed using GraphPad Prism 8.0 and Image *J* statistical software. Unpaired *t*‐test and one‐way ANOVA were used to compare differences between groups. *P* < 0.05 was considered statistically significant.

## 3. Results

### 3.1. Cartilage Damage Not Improved After Interventions

**FIGURE 2:**
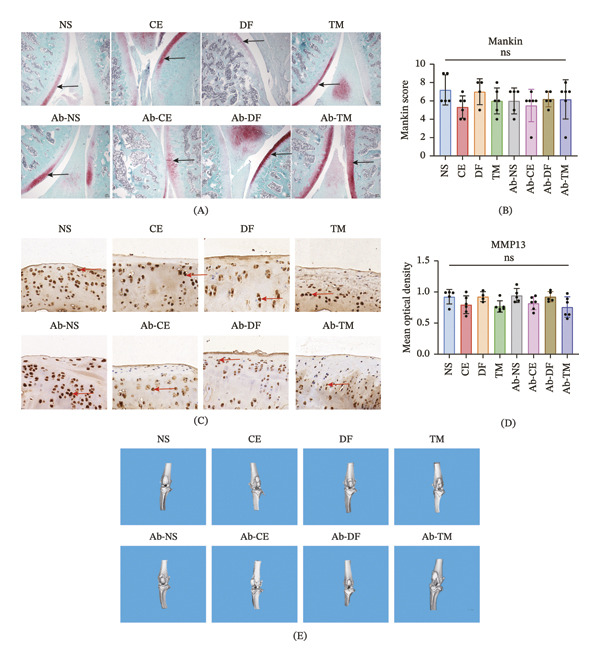
Changes in knee joint structure in rats. (A) Histological images of knee joints (original magnification, × 4). (B) Quantitative analysis of articular cartilage damage using Mankin’s scores. (C) Immunohistochemical staining for MMP13 in articular cartilage (original magnification, × 100). (D) Quantitative analysis of MMP13 expression in cartilage. (E) Three‐dimensional CT reconstruction images for evaluating osteophyte formation. Black arrows indicate articular cartilage; red arrows indicate areas of dye enrichment.

OA was characterized by deterioration of the articular cartilage. We used Senna solid green staining to mark the cartilage damage in the knee of the rats and the Mankin scoring criteria to determine the effectiveness of the three drugs on ACLT‐induced OA. The pharmacologic intervention did not show a significant chondroprotective effect when compared to NS (Figure 2A–D), although there was a trend for slightly reduced severity of OA in the CE group (Figure 2A and B). Comparing the articular cartilage of rats in the CE group to that of rats treated with NS revealed similar results in immunohistochemistry. However, none of the treatments (CE, DF, and TM) were able to significantly lower the expression of MMP13 in cartilage (Figure 2C and D). In addition, the development of bony encumbrances was an important pathological change during the progression of OA. To this end, we reconstructed the whole knee joint of rats using CT three‐dimensional image reconstruction, and the results showed that the pharmacological interventions had no discernible effect on the formation of bony encumbrances (Figure 2E).

### 3.2. Systemic and Localized Joint Inflammation Reduced After Interventions

**FIGURE 3:**
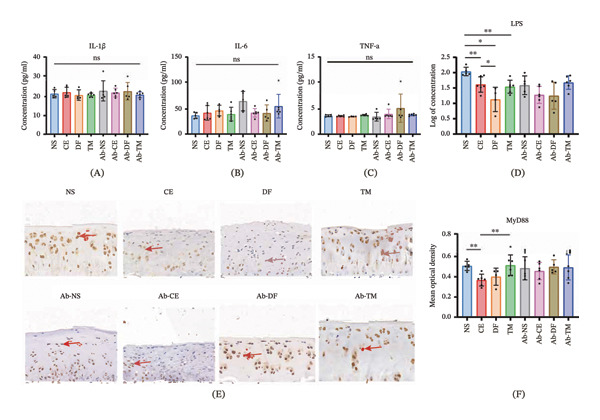
Changes in related tissue structure and inflammatory indicators in rats after drug intervention. (A–D) Serum levels of inflammatory cytokines, including (A), IL‐1β; (B), IL‐6; (C), TNF‐α; and (D), LPS. (E). Immunohistochemical staining for MyD88 in articular cartilage (original magnification × 100) (F) Quantitative analysis of MyD88 expression in cartilage. Red arrows indicate areas of positive staining (dye enrichment). ^∗^ *P* < 0.01.

In serum, variables associated with inflammation included IL‐1β, IL‐6, TNF‐α, and LPS. The three drugs, especially DF, significantly reduced serum LPS levels but not IL‐1β, IL‐6, or TNF‐α concentrations (Figure 3A–D). This effect disappeared when the antibiotics were taken. After LPS, MyD88 was the key junction molecule. Immunohistochemistry revealed a substantial drop in MyD88 expression in the articular cartilage of rats in the CE group (*P* = 0.0087) as well as a trend of a significant decrease in the DF group (*P* = 0.0635). Additionally, there was also a statistically significant decrease in MyD88 expression in the CE group as compared to the TM group (*P* = 0.0087), whereas there was only a slight tendency for MyD88 expression in the DF group to decrease as compared to the TM group (*P* > 0.05). However, after pseudo‐germ‐free treatment, no significant difference in the expression of MyD88 within articular cartilage was observed between the groups (Figure 3E and F).

### 3.3. Pain in Rats Relieved After Intervention

**FIGURE 4:**
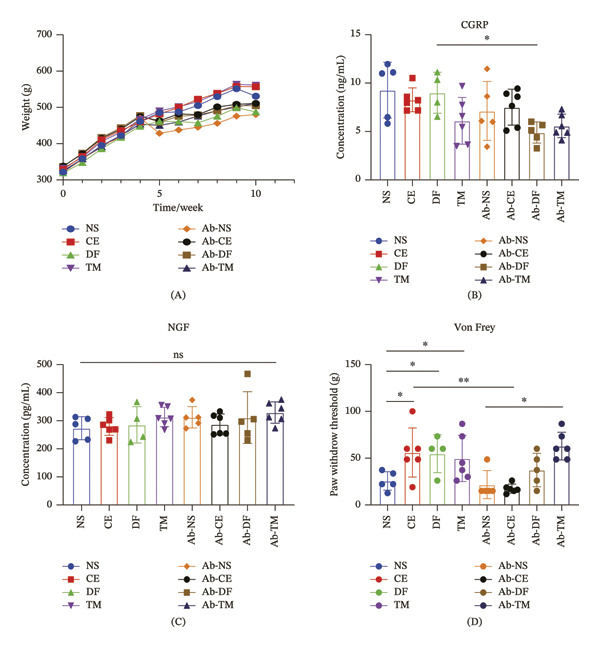
Changes in pain‐related indicators in OA rats following drug intervention. (A) Dynamic changes in body weight. (B). Serum levels of CGRP. (C) Serum levels of NGF. (D) Paw withdrawal threshold. ^∗^ *P* < 0.01.

Though the rate of weight gain was lower in the DF group and the antibiotic treatment groups, there were no significant differences in body weight among the groups of rats throughout the experiment (Figure 4A). NGF and CGRP are both factors related to pain, and there were no significant differences found in the serum concentrations of NGF and CGRP among the eight groups of rats (Figure 4B and C). Finally, we conducted a pain behavior test in rats at the end of the experiment to confirm the efficacy of CE, DF, and TM in reducing arthritis pain. According to the Von Frey test, the three drugs showed similar analgesic effects, but differences appeared after antibiotic treatment. There was no significant change in PWT of rats in the Ab‐CE and Ab‐DF groups compared with the Ab‐NS group, while the PWT of rats in the Ab‐TM group was significantly higher than that in the Ab‐NS group (Figure 4D).

### 3.4. Effects of Pharmacologic Interventions on the Gut Microbiome and Their Metabolites, SCFAs

**FIGURE 5:**
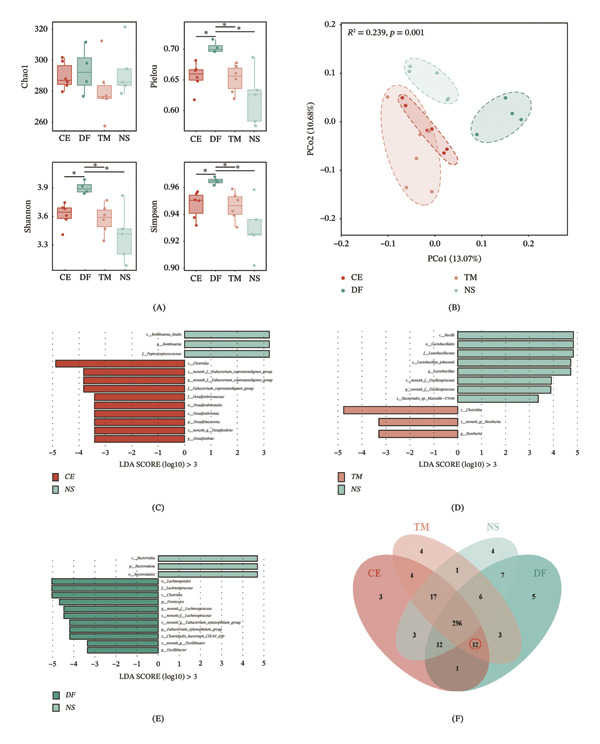
Modulation of gut microbial community structure and composition in response to drug interventions. (A) Alpha‐diversity analysis of the gut microbiota. (B) Beta‐diversity analysis of the overall bacterial community structure. (C–E) LEfSe analysis identifying differentially abundant key bacterial genera between the NS group and CE, TM, and DF groups, respectively. (F) Venn diagram showing the overlapping and unique differential gut microbiota among experimental groups. ^∗^ *P* < 0.05.

16S rRNA gene sequencing was used to identify the gut microbiome in the feces of rats in the NS, CE, DF, and TM groups at the end of the experiment. The Chao1 index did not significantly differ among the groups. However, the Pielou index, Shannon index, and Simpson index were significantly higher in the DF group than the other three groups, and the CE and TM groups also showed a significant uptrend when compared to the NS group (Figure 5A). Following the drug intervention, PCoA revealed that the gut microbiome of the rats in the CE, DF, and TM groups differed significantly from that of the NS group (Figure 5B). Intestinal microbial community structure was analyzed with LEfSe to select the genera that significantly contributed to the differences in gut microbiota among the groups. The abundance of *Eubacterium* and Desulfovibrionaceae in the CE group was significantly higher than that in the NS group, and the abundance of Lachnospiraceae, *Eubacterium*, and *Oscillibacter* in the intestinal tracts of the DF‐treated rats was significantly higher. In the TM group, *Roseburia* was marked enriched in the intestines of rats (Figure 5C–E). The Venn diagrams were used to characterize the commonality of gut microbiota changes after treatment with the three drugs. More than half of the 12 homogeneous genera that we found to be enriched in rats in the CE, DF, and TM groups (Figure 5F) belonged to the SCFA‐producing bacteria families Oscillospiraceae and Lachnospiraceae. Details are provided in Supporting Table 1.

**FIGURE 6:**
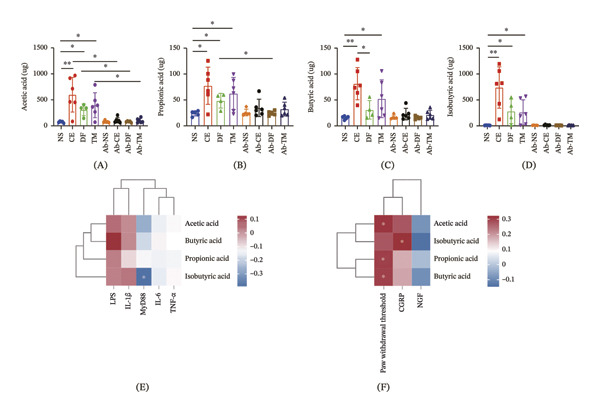
Effects of drug intervention on fecal SCFA profiles and their correlations with inflammatory and pain‐related indicators. (A–D) Fecal concentrations of (A) acetic acid, (B) propionic acid, (C) butyric acid, and (D) isobutyric acid. (E) Correlation analysis between SCFAs and indicators of inflammation. (F) Correlation analysis between SCFAs and indicators of pain. ^∗^ *P* < 0.01.

The SCFAs in the feces were measured using GC–MS. The concentrations of acetic acid, propionic acid, butyric acid, and isobutyric acid in the feces of rats in the CE and TM groups were significantly higher than those in the NS group (Figure 6A–D). Similarly, the rat’s feces in the DF group contained significantly higher levels of acetic acid, propionic acid, and isobutyric acid than those in the NS group. The butyric acid also showed a significant upward trend (*P* = 0.1111) compared to that of the rat’s feces in the NS group, though the difference was not statistically significant.

Furthermore, we analyzed the correlation between pain and inflammation indicators and SCFAs, respectively. We found that the concentrations of acetic acid, propionic acid, and butyric acid in feces were positively correlated with PWT, and the concentration of isobutyric acid was negatively correlated with the concentration of MyD88 in joints (Figure 6E and F).

### 3.5. Correlation Analysis Between the Gut Microbiome and Clinical Indicators

**FIGURE 7:**
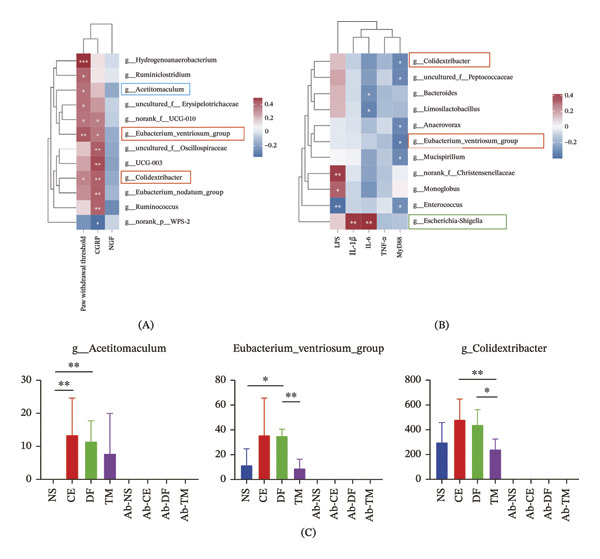
Correlation between gut microbiota and pain‐related indicators. (A) Heatmap showing correlations between gut microbiota and pain‐related indicators. (B) Heatmap of correlations between gut microbiota and pain‐related indices. Genera simultaneously positively associated with pain relief and negatively correlated with inflammatory markers are highlighted in red frames. Among the 12 key bacterial genera, those positively correlated with pain reduction are marked in blue frames, and inflammation‐associated genera are labeled in green frames. (C) Relative abundance of several key bacterial genera. ^∗^ *P* < 0.05, ^∗∗^ *P* < 0.01.

We analyzed the correlation between the gut microbiome, inflammation indicators, and pain. The serum concentration of LPS was shown to have a negative correlation with *Enterococcus* (*P* < 0.01) but a positive correlation with *Monoglobus* (*P* < 0.05) and a positive correlation with *Norank_f__Christensenellaceae* (*P* < 0.01). The abundance of *Escherichia-Shigella* was observed to substantially positively correlate with serum concentrations of IL‐6 and IL‐1β (*P* < 0.01). Among the 12 coenriched genera, *Acetitomaculum* showed a positive correlation with PWT. Furthermore, we discovered that in both cases, *Eubacterium ventriosum* and *Colidextribacter* were negatively correlated with intra‐articular MyD88 expression and positively correlated with PWT (*P* < 0.05) (Figure 7A and B). Comparing the abundance of these bacteria, we found that the abundance of *Acetitomaculum* was significantly higher in the CE and DF groups than in the NS group, and the abundance of *Eubacterium ventriosum* and *Colidextribacter* also increased significantly (Figure 7C).

## 4. Discussion

OA is prevalent among middle‐aged and elderly individuals, marked by the gradual degeneration of cartilage, leading to persistent joint pain and dysfunction [21]. The inadequacies of current treatment options underscore the necessity for novel pharmaceutical interventions. The involvement of low‐grade inflammation, modulated by innate immunity, is pivotal in the pathogenesis of OA [4]. Considering the significant impact of the gut microbiome on inflammation and immunity, targeting the gut microbiome may offer a promising therapeutic strategy. Our study revealed the potential role of the gut microbiome in the efficacy of NSAIDs in the treatment of this disease through antibiotic‐induced gut microbiota depletion in a rat OA model. Although antibiotic treatment may exert wide‐ranging systemic effects on the host, such nonspecific effects can complicate the interpretation of experimental results to a certain extent, meaning the observed effects cannot be entirely attributed to the absence of the gut microbiota. Notably, since all experimental and control groups in this study received identical antibiotic pretreatment, the general systemic effects of antibiotics were largely consistent across groups and thus partially minimized during comparative analyses. Even so, potential off‐target effects of antibiotics independent of gut microbiota depletion cannot be entirely excluded. Nevertheless, this approach allows us to make a preliminary assessment of the impact of the gut microbiota. In our study, NSAIDs have the capacity to alter the composition of intestinal microbiota, and the metabolites of the drug‐modified gut microbiota fatty acids may be potential mediators of the analgesic effect of the drugs.

### 4.1. Analgesic Effects of Compounds CE, DF, and TM

Joint pain, a primary clinical symptom of OA, was the primary motivator for patients seeking medical care. Localized inflammatory changes in the affected joints, central and peripheral nociceptive hypersensitivity, and individual patient factors such as metabolic syndrome and genetic predisposition collectively contribute to the chronic pain experienced by individuals with OA [22]. Our research findings indicate that Compounds CE, DF, and TM demonstrate efficacy in alleviating pain in rats, with the analgesic properties of CE and DF notably diminished following antibiotic treatment, underscoring the potential influence of the gut microbiome on the pharmacological actions of these two drugs. However, the analgesic effect of TM still exists after antibiotic treatment, which can be explained by its central analgesic mechanism acting independently of intestinal microbe [23]. In other words, its regulatory effects on LPS levels and SCFAs associated with gut microbiota may represent secondary effects or relevant changes occurring during the drug’s systemic action.

### 4.2. Regulation of Inflammatory Response

It was observed that the concentrations of IL‐1β, IL‐6, and TNF‐α in the rats belonging to the CE, DF, and TM groups did not show significantly different changes compared to those in the NS group, potentially attributed to the timing of blood collection. Following acute ACL injury, inflammatory factors gradually decrease to baseline levels in patients [24].

Notably, while serum levels of classic proinflammatory cytokines (IL‐1β, IL‐6, and TNF‐α) did not differ significantly among groups, we observed marked changes in systemic LPS. This finding aligns with the paradigm of OA as a condition of low‐grade, chronic inflammation. In such a state, fluctuations in downstream effector cytokines in the periphery can be transient and may not stably reflect the local joint environment. In contrast, endotoxemia resulting from a compromised intestinal barrier represents a more persistent, upstream driver of systemic inflammation. LPS can act as a pathogen‐associated molecular pattern (PAMP) to enter the joint via blood circulation and bacterial translocation, activating Toll‐like receptor 4 on the surface of synovial cells and macrophages, thereby chronically initiating innate immune signaling in joint tissues [25], potentially explaining the observed reduction in local MyD88 expression following treatment, even in the absence of significant changes in circulating cytokines. Thus, LPS may serve as a more sensitive and stable biomarker of the systemic inflammatory tone in chronic OA compared to individual cytokines, warranting further investigation.

Our finding indicates that compared with the NS group, the concentration of LPS in the CE and DF groups was significantly reduced, and the MyD88 expression level in the joints of these two groups of rats showed a similar pattern. This may be due to the decreased activation of Toll‐like receptor 4 in the joint caused by the decreased systemic LPS level.

Notably, this trend was not observed in the antibiotic treatment group, indicating that CE and DF might also exert alleviating effects on local inflammation, potentially associated with the inhibition of the LPS–TLR4–MyD88 pathway. However, this study did not directly detect or verify all key molecules within this pathway. Subsequent research is warranted to further elucidate the specific role and regulatory mechanisms of the LPS–TLR4–MyD88 pathway in this process through specific intervention experiments and other assays.

It is worth noting that the down regulation of MyD88 expression in cartilage tissue may merely reflect the weakening of local immune signaling pathways. Correlation analysis reveals no significant linear relationship between MyD88 levels and PWT, indicating that there is no direct mechanistic correlation between the reduction of local inflammation in the joint and the observed analgesic effect.

### 4.3. SCFAs: Potential Mediators of Drug Analgesia in NSAIDs

The relationship between the gut microbiome and the host immune system is reciprocal. The gut microbiome played a crucial role in promoting the maturation and balance of the immune system, as well as in providing protection against pathogenic organisms. Conversely, inflammation resulted from a dysregulated immune response can impact the composition of the gut microbiome [26]. This dynamic interaction was also relevant in the context of OA [27, 28], where the metabolites were produced by the gut microbiome. SCFAs, derived from the glycolysis of carbohydrates by the gut microbiome, were integral in preserving the intestinal barrier, suppressing tumor growth, and modulating inflammation and energy metabolism [29, 30]. Researches have demonstrated that these SCFAs can modulate cytokine production, promote immune cells differentiation, and inhibit neutrophil chemotaxis through *G* protein–coupled receptor (GPCR) activation [31–33]. SCFAs also inhibit the PI3K/Akt/GSK‐3 *β* signaling pathway by suppressing histone deacetylase activity, while suppressing the inflammatory response of microglia to participate in the epigenetic regulation of chronic pain. In addition, SCFAs can activate the vagus nerve to regulate pain perception [33]. Moreover, SCFAs have been found to alleviate neuropathic pain, with studies indicating that elevated levels of these SCFAs can mitigate nitroglycerin‐induced hypersensitivity and reduce pain perception [34]. In this study, pharmacological intervention with Compounds CE, DF, and TM significantly elevated fecal concentrations of SCFAs in rats. Critically, antibiotic‐induced ablation of the gut microbiota substantially diminished the analgesic response to NSAIDs, implicating a functional role for the microbiota in NSAID‐mediated pain relief. Subsequent correlation analyses demonstrated that fecal levels of acetic acid, propionic acid, and butyric acid were positively associated with mechanical pain thresholds. Building on established evidence that SCFAs exert anti‐inflammatory effects and reduce the excitability of pain neurons, we speculate that SCFAs, the metabolites of gut microbiota, may be important molecules involved in the analgesic effect of NSAIDs. Although we did not detect the content of serum SCFA due to sample limitations, combined with the reduction of fecal SCFA in rats after the depletion of pseudoaseptic gut microbiota in this study, the analgesic effect of NSAIDs was weakened, which still supports the potential mechanism that SCFAs may be involved in the analgesia of NSAIDs.

Furthermore, our study revealed several potential advantageous bacterial genera through correlation analyses. Specifically, we observed that the abundance of *Acetitomaculum*, which was found to be enriched in the CE, DF, and TM groups, exhibited a positive correlation with PWT. Additionally, *Eubacterium ventriosum* and *Colidextribacter* were positively associated with pain alleviation and inversely related to makers of intra‐articular inflammation. Notably, both genera were associated with heightened production of SCFAs [35–37].

### 4.4. Limitations

Our study has revealed a significant contribution of the gut microbiome to the efficacy of NSAIDs in therapy. SCFAs, the products of gut microbiota, may play a role in NSAIDs drug treatment. Although our study did not directly confirm the reducing effect of SCFAs on arthritis pain, we established the relationship between the gut microbiota, SCFAs, and NSAID analgesia.

However, there remain pressing inquiries that require attention. Specifically, the attrition of animals during the experimental timeline resulted in a smaller final sample size than initially planned. Although our statistical analysis showed significant results for the primary endpoints, the limited sample size increases the risk of Type II errors and reduces the power to detect more subtle effects or to conduct comprehensive subgroup analyses. Future studies with larger cohorts are warranted to elucidate the causal relationship between the analgesic effects of SCFAs and NSAIDs. Secondly, the choice of the age of rats is also a matter of concern. In this study, young rats were selected to construct the OA model, and OA and knee pain are mostly seen in adult and elderly people in clinic. The structure of the intestinal microbiota in the elderly is affected by age, sex, aging, and other related physiological changes, which is significantly different from that of young rats. Moreover, elderly patients with OA often have a variety of basic diseases, and their pathological state is more complex. Therefore, this study selected young male rats for the experiment. Although this can minimize the interference of age‐related and gender‐related confounding factors on the gut microbiota and clearly reveal the direct association between NSAIDs, the gut microbiota and joint inflammation, the statistical power of the research results may be affected to a certain extent, and it cannot fully simulate the effects of NSAIDs on OA patients and the regulatory characteristics of the gut microbiota. Future studies will select adult or naturally aging OA rat models, close to the pathological state of clinical middle‐aged and elderly patients, and include male and female animals for further research to verify the regulatory effect of NSAIDs on gut microbiota and the impact of gut microbiota on OA treatment.

Furthermore, this study employed broad‐spectrum antibiotics to establish a pseudo‐sterile model for verifying the role of the microbiota. Although antibiotic‐mediated depletion of the microbiota remains a prevalent and efficacious approach for confirming the causal involvement of the gut microbiota, the process of depleting the microbiota with antibiotics may concurrently influence the body’s immune function, inflammatory signaling pathways, and systemic metabolic homeostasis. These influences may independently impact cartilage metabolism, pain perception, or the inflammatory state of joints. In the future, the adoption of more precise microbiota modulation strategies, such as targeted bacterial elimination or germ‐free animal models, will facilitate a clearer understanding of the role of the gut microbiota in NSAID drug therapy.

## 5. Conclusion

In summary, our work provides conclusive evidence for an association between the gut microbiome modified by NSAIDs and OA. Our study suggests that although NSAIDs cannot attenuate cartilage damage and slow the progression of OA, the drug‐modified gut microbiota may potentially participate in the analgesic effect of drugs through the production of SCFAs, whether it is a nonselective COX inhibitor DF or a COX‐2 selective inhibitor CE. In addition, we also found that NSAIDs may inhibit the activation of LPS‐related genera and the LPS/TLR4/MyD88 pathway, thereby reducing systemic and local joint inflammation, although this still needs further research to confirm. In addition, we have identified several possible beneficial bacteria for joints. This study provides a new theoretical basis and practical guidance for the clinical treatment of OA.

**Nomenclature**
NSAIDs: Nonsteroidal anti‐inflammatory drugs
ACLT: Anterior cruciate ligament transection
NS: Saline
CE: Celecoxib
DF: Diclofenac
TM: Tramadol
CGRP: Calcitonin gene–related peptide
NGF: Nerve growth factor
OA: Osteoarthritis
PWT: Paw withdrawal threshold
TBS: Tris‐buffered salt solution
SCFA: Short‐chain fatty acid
LPS: Lipopolysaccharide

## Author Contributions

Xuesong Wang: conceptualization, methodology, and writing–review and editing. Hao Meng: investigation, formal analysis, and writing–original draft. Baoshan Yuan and Yongning Dong: investigation and writing–original draft. Dongsong Liu: methodology, investigation, and writing–original draft. Feng Zhang: project administration, formal analysis, and writing–review and editing. Hong Cao: supervision, project administration, and writing–review and editing. Dan Li, Ju Yang, Jing Sun, and Yingyu Wang: resources, formal analysis, and writing–review and editing. Bingqian Chen: supervision and writing–review and editing. Jiai Yan: formal analysis, data curation, and writing–review.

## Funding

This work was supported by the grants of Top Talent Support Program for Young and Middle‐Aged People of Wuxi Committee of Health (BJ2023052), the National Natural Science Foundation of China (82370809; 32101033), the Natural Science Foundation of Jiangsu Province (BK20210060), the Key Research Project of Health Commission of Jiangsu Province (K2023004), Key Discipline Construction Program of Wuxi Commission of Health (CXTD2021003), and Wuxi Science and Technology Bureau, “Taihu Light” Science and Technology Research Program (K20253003).

## Conflicts of Interest

The authors declare no conflicts of interest.

## Supporting Information

Additional supporting information can be found online in the Supporting Information section.

## Supporting information


**Supporting Information** Supporting Information is freely available. Table S1 presents 12 common OTUs identified in CE, DF, and TM groups, while Figure S1 shows the LEfSe analysis and comparison between TM and NS groups.

## Data Availability

The data that support the findings of this study are available on request from the corresponding author. The data are not publicly available due to privacy or ethical restrictions.
