# Supplementary figures and images for: Short‐Chain Fatty Acids as Potential Mediators of NSAIDs’ Effects on Arthritis Pain Relief
Source: Pain Res Manag. 2026 Jun 26;2026:9190926. doi: 10.1155/prm/9190926 (PMC13307179; doi:10.1155/prm/9190926)

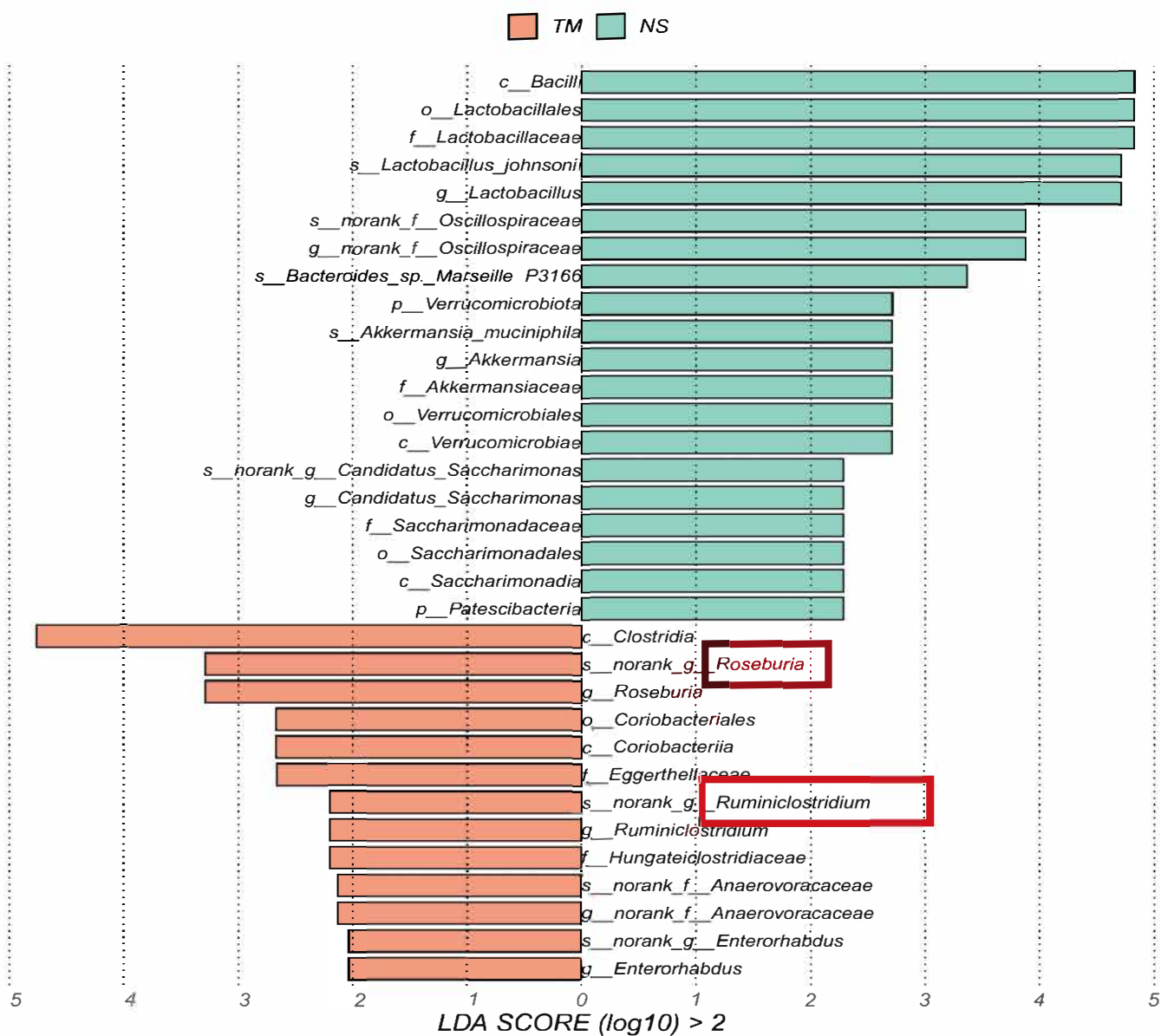

Supplementary Figure1 Lefse analysis and comparison between TM group and NS group

Supplement: Supplementary file 1 — Supporting Information Supporting Information is freely available. Table S1 presents 12 common OTUs identified in CE, DF, and TM groups, while Figure S1 shows the LEfSe analysis and comparison between TM and NS groups. [file PRM-2026-9190926-s001.zip › Supplementary Fig 1.pdf]
